# Supplementary figures and images for: Towards distortion-free imaging of the eye
Source: PLoS One. 2021 Jun 10;16(6):e0252876. doi: 10.1371/journal.pone.0252876 (PMC8192003; doi:10.1371/journal.pone.0252876)

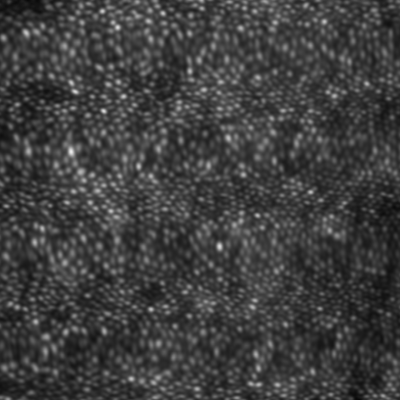

Supplement: S1 Fig — Example shows 100 contiguous frames from the cluster of 1000 frames corresponding to Fig 8A. (TIF) [file pone.0252876.s001.tif]
