# Supplementary material for: Towards distortion-free imaging of the eye
Source: PLoS One. 2021 Jun 10;16(6):e0252876. doi: 10.1371/journal.pone.0252876 (PMC8192003; doi:10.1371/journal.pone.0252876)
Supplement: Attachment — Submitted filename: Response to Reviewers.docx [file pone.0252876.s003.docx]

We thank the reviewers and editor for their time and constructive comments, which have helped us to improve the clarity of the manuscript. Please find pertinent comments reproduced below in *italics*, with our replies following in plain text and passages from the manuscript reproduced in “quotations” with new additions marked in red.

**Additional Journal Requirements:**

*1. Please ensure that your manuscript meets PLOS ONE's style requirements, including those for file naming. The PLOS ONE style templates can be found at*

*https://journals.plos.org/plosone/s/file?id=wjVg/PLOSOne_formatting_sample_main_body.pdf and*

*https://journals.plos.org/plosone/s/file?id=ba62/PLOSOne_formatting_sample_title_authors_affiliations.pdf*

We have reviewed these documents and believe the manuscript now follows the journal formatting requirements. This includes changes to the title page, headings/subheadings, figure references, equations, and Table 1.

*2. If materials, methods, and protocols are well established, authors may cite articles where those protocols are described in detail (for instance here, Bedggood P, Metha A. De-warping of images and improved eye tracking for the scanning laser ophthalmoscope. PloS one. 2017;12(4):e0174617), but the submission should include sufficient information to be understood independent of these references*

We have added a new Methods section titled “Dynamic correction method” whose contents are as follows:

Line 425: “Although described in full elsewhere, it is worthwhile to give a brief overview of our previously published method for correcting dynamic distortions (10). These are generated dynamically in raster-based imagery because eye movements occur during the sequential acquisition of image points. The method relies on the assertion that, given the stochastic nature of fixational eye movements, the expected value of distortion “seen” by one part of the retina should not differ from that expected for any other part of the retina. In other words, the bias in apparent displacement for each piece of tissue should trend towards zero when many frames are considered. Accordingly, the local distortions accrued within any particular frame can be discovered by registering each piece of tissue therein across a large number of frames; if it is found that the average displacement for that tissue differs from zero, this must result from the distortion inherent in the frame in question, such that the net bias does sum to zero.

The assumption of zero bias described above must be violated in the case of systematic error which produces static distortion, present on every frame. In that case, assuming that the bias is zero should be expected to result in the full degree of underlying systematic bias remaining within the “recovered” frame. This underpins the strategy advanced in the current work, whereby dynamic recovery is first applied in order to produce a handful of frames which all suffer from a common systematic bias.”

**Reviewer #1: Review of PONE-D-20-39508**

*However, the principle of the method is not well written, and all simulation parameters are not well organized. There are some questions in the result of correction for systematic and dynamic distortions (LINE 418-479). These points are required to be addressed before publication.*

Thank you for the feedback. We believe we have now addressed each of these points as detailed below.

*- Classification of distortions*

*The word "systematic distortion" sounds to me that static distortions due to an imaging instrument. In fact, the authors use "dynamic distortions" as a counterpart. But then the "pseudo-systemic distortion" (LINE 27) seems odd. Because "DIOS" and "dynamic restoration methods" are image-based heuristics, introducing another classification based on the type of image distortions, such as "static image distortions" and "dynamic image distortions," may help clarify the discussion of results.*

This is a valid comment. We have now replaced all mentions of “systematic” distortion, with “static” distortion. The word “systematic” now appears in only one place (Introduction, Paragraph 3) when listing examples of static distortion.

*-Methods*

*--Characteristics of imaging modalities*

*The authors did the proof of concept of their idea, DIOS. The images without distortions are used as ground truth, and scanning ophthalmoscope images are simulated by distorting them. To obtain the "original" ground truth images, the authors used a flood-illumination AO ophthalmoscope.*

*The main concern is whether the different imaging characteristics between flood-illumination-based and scanning-based ophthalmoscopes affect the performance of the presented method.*

*For example, speckle appearance might be different between them. The images of the flood illumination AO ophthalmoscope exhibit less speckle. However, an image of single frame of scanning ophthalmoscope may express more speckle.*

*For the readers to properly understand the possible difficulties, these points should be discussed, and the details of the system should be described, although the previous authors' paper [10] is cited.*

We did not in fact intend for simulated images to be considered as being from a scanning-based or a flood-based system per se. Considerations such as speckle or other sources of deviation between the quality of flood and scanning instruments would be difficult to include.

We have clarified as follows in the Methods section:

Line 348: “As in our previous work, the ground truth data were used here to simulate imagery acquired in the presence of distortion. The fidelity of distortion recovery was assessed by calculating the Pearson correlation in image intensity between the recovered and ground truth images. Applied distortion was either static alone, which could be produced by a “flood” illuminated system or by a scanning system operating at high frame rate or on a stationary eye; or a combination of static and dynamic distortion, which could be encountered with a scanning instrument in the presence of fixed sources of distortion. It should be noted that our simulation does not consider common differences which may be found between flood or scanning image modalities, for example regarding shot noise or speckle characteristics which are not expected to affect distortion *per se*.”

It is also worth noting that any such intensity variations are not relevant to the algorithm presented, other than that they could have predictable effects on registration quality. Where they do not affect registration quality, the recovered distortion maps cannot be affected, because the algorithm only considers registration information. We have added this to the Discussion in the following section:

Line 609: “Any correction applied to real images must use real image registration, which will be subject to some degree of error. Although the least squares nature of the solution is likely to be resilient to such errors, we have explored this only briefly (see Supplementary Information 1), wishing here primarily to demonstrate in principle that the method itself does not impose any significant limitation to accuracy. Note that consideration of error due to inaccurate image registration encompasses errors which may arise due to frame-to-frame variations in intensity. Such variations could be caused by factors such as light-evoked fluctuations in reflectance or shot noise or the oxygenation state of blood, etc. Where these do not cause error in image registration, such intensity variations will not affect the quality of the recovered distortion maps, because the algorithm presented operates only with registration information rather than intensity information.”

We have also provided further details of the imaging setup as recommended:

Line 329: “As in our previous work (10), “ground truth” images were obtained from by imaging the cone photoreceptor mosaic in a young, healthy subject at high speed (200 fps; frame exposure 2.5 ms) with a flood-illuminated adaptive optics ophthalmoscope and an imaging wavelength of 750 ± 25 nm. Images were acquired over a 7.5 mm pupil after adaptive optics had reduced the root-mean-square (RMS) wavefront error to 0.05 µm or less, i.e. below the diffraction limit. The ground truth data were montaged from multiple imaging locations temporal and within 2° of the foveal centre. This allowed a relatively wide field to be imaged without noticeable degradation in quality towards the edge of the field. Images of the photoreceptor mosaic in general provide an excellent example…”

*--DIOS principle*

*Overall the principle of DIOS is quite difficult to understand. Equations are not well written, and there is no consistency among equations, figures, and tables.*

Thank you for the specific comments given below, which have helped us to clarify our descriptions. Please see below for changes made regarding this issue.

*--- Line 96*

*What does the symbol "T" mean? Although the description appears later (Line 136), it should be explained where the symbol first appears.*

We have amended as advised:

Line 139: “Consider the 1D scene shown in Table 1 containing letters of the alphabet “E” through “K”, spaced one unit apart. The scene was translated by an amount “T” pixels in the x dimension by either T = 0 units…”

*---Line 117*

*What does mean "the reference image (xR)" and "the moving image, xM"? According to the description around Equation 1, it might be the locations of the same object in the reference image and the moving image.*

We have added the following clarification by changing our phrasing “in terms of” to “in the co-ordinate system of”, which now gives a clear meaning for the terms xR and xM:

Line 157: “…Returning to the task of determining the distortion map, the object space co-ordinates (xO) of a given feature can be expressed independently in the co-ordinate system of the reference image (xR) and in the co-ordinate system of the image to be registered (the moving image, xM):”

*---Line 119-120*

*The numbers (2, 1, 0, 3, -3) suddenly appear. Please explain the details. Do the authors use "image 1" as "Reference image" and "image 2" as "Moving image"?*

These numbers are evaluations of the given equations in the specified example. We have clarified by including an extra step as follows:

Line 160:

“Reference image:	$x_{O}=x_{R}+f(x_{R})- T_{R}$

Moving image:	$x_{O}=x_{M}+f\left( x_{M} \right)- T_{M}$

In this particular example (i.e. for the feature H), these expressions evaluate as follows:

Reference image:	$x_{O}=2+1- 0= +3$

Moving image:	$x_{O}=0+0-(-3)= +3$

*---Line 124*

*From two formulae ("Reference image" and "Moving image"), Equation 1 cannot be derived. The symbol "T" should disappear. Perhaps the authors should discriminate it for the reference image and moving image as "TR" and "TM".*

The reviewer is correct, these “T” terms were not intended to be the same. The revised equations reproduced above now designate T_R_ and T_M_. Thank you for bringing this to our attention.

*---Figure 1*

*Figure 1 is unclear. What is the vertical axis? What do the labels "f(x1)", "f(x2)", and "f(x3)" mean? What do the circles mean?*

We have now redrawn Figure 1 including axis labels, which we reproduce below. The figure is now simplified to consider just 2 example points, and the comparison being made now further indicated by the dashed arrows. The figure caption has also been re-written as follows, which neatly encapsulates the method:

Line 192: “**Fig 1. Illustration of a paired functional difference**. An example static distortion map f(x) is defined over the pixels of the detector (x). Circles represent examples where detector pixels (x1, x2) were determined, by image registration, to have “seen” the same object in different frames. The value of the distorting function at the two locations is f(x1), f(x2). The object in question has shifted between moving and reference frames by an amount (DM,R), which is equivalent to the sum of a) the difference in the distorting function f(x2) – f(x1), and b) the overall translation of the scene (e.g. T2 – T1, or simply T2 if the position of the reference frame is defined as zero). Therefore, pixel-wise registration between shifted versions of the same scene can be used to build up information about the shape of the distorting function that is common to each frame.”

*---Line 215*

*The description of "anti-aliasing" is too short. Please describe more details, or please cite literature if there are.*

*The reason why the anti-aliasing can achieve a precision similar to upscaling is unclear.*

We have replaced the hard-to-prove statement regarding “precision similar to upscaling”, with a statement to the effect that fractional displacements were well-handled.

In addition we have now given the equation required to calculate the pixel weightings (and note that it is also implemented in the example code supplied in Supporting Information):

Line 264: “In principle this problem could be solved to arbitrary precision by upscaling the images, however this is impractical because the solution is already very resource intensive. Instead, we implemented a form of 2x2 anti-aliasing which produced satisfactory handling of fractional displacements. Under this scheme, the negative entries in the coefficient matrix, which were -1 in the simplified example, are distributed between the 4 pixels bounding the fractional pixel address, weighted by their proximity to that address. The sum of the coefficients for these 4 pixels remains -1, and the “reference” pixel is left unchanged at +1. The appropriate pixel weightings for each of the 4 bounding pixels were calculated by the product of independent weightings for x and y proximity, as follows:

W(xa,yb) = (1 – |x – xa|) * (1 – |y – yb|)

The weightings calculated this way sum to unity for the 4 pixels, and dictate the proportion of the given object’s intensity that will be recorded in each of the 4 locations.”

*---Equation*

*Where is Equation 5?*

Thank you for pointing out this oversight. We have now renamed Equation 6 and all references to it, to Equation 5.

*--Line 312*

*What are 2D sine functions? Equation 6 describes two 1D sine functions. Do the authors mean that both Dx and Dy are distributed in the xy-plane? If it is, Equation 6 should be corrected.*

Thank you for picking up another oversight. The equation has now been corrected to show that Dx and Dy are each functions of x and y (the equation is now numbered Equation 5 as just described).

*-Results*

*--Systematic distortion, dynamic distortion, and eye movements*

*There are many parameters in simulations of distortions and eye movements. It is difficult to follow the logic and interpret the results.*

*For each result, all simulation parameters and constraints must be explicitly described; otherwise, the results cannot be interpreted correctly. There are many ambiguities in the current manuscript. For example, the constraints to translation (eye movements simulation?) described from the Line 400 to Line 404 are commonly applied to all results?*

*In my opinion, the parameters and constraints of the simulation should be listed in the "Methods" section since they are the parameters of the "Verification Method."*

The reviewer here makes two valid points:

- Less ambiguous specification of model parameters is required in some places
- This information should be placed in the Methods, not the Results

In regards to the point regarding less ambiguous information, we believe that the more complicated modelling represented in Fig 6 and Fig 7 are likely causes of confusion. Hence we have placed the relevant information in the Methods as suggested in the second point, and stipulated that certain parameters apply specifically to particular Figures, as follows:

FIG 6

Line 498: “To demonstrate that the method can handle distortions of arbitrary form, we modelled 1,000 static distorting functions whose parameters (Equation 5) were drawn uniformly from the full range described above (see section “Simulating distortion”). Given the computation requirements for larger arrays, here we recovered only the central 100x100 pixel region. The simulation parameters supported extreme distortions (the most distorted example is shown in Fig 6A). For each simulated run shown in Fig 6, we “acquired” 4 frames with the eye having been translated and rotated by random amounts (see section “Simulating full-field movements”).”

The referred-to Methods sections read:

Line 391 (Simulating distortion): “There were accordingly 6 parameters set for each distorting function. In the Results presented below, the amplitude parameters lay in the range ± 5 pixels, the frequency parameter in the range 0 to 5 cycles, and the phase parameter in the range 0 to 2π.”

Line 376: (Simulating full-field movements) “Since one cannot select what movements a real eye undergoes, we tested the new method with a large number of simulated runs, with the parameters for rigid body movement randomly drawn on each run. For this exercise the translation parameters were chosen from the range ± 10 pixels and the rotation parameter ± 5°. As described above under “Constraining the Solution”, it is necessary to ensure that images selected contain “unique” displacement information. We therefore applied somewhat arbitrary partial constraints to the translation of the retinal scene: translations each differed in magnitude by at least 2 pixels, and in direction from the origin by 45° or more. These constraints are by no means optimal, but could serve as an initial guide for selecting images from real data sets.”

FIG 7

Line 518: “The DIOS procedure may be applied in sequence with the dynamic recovery procedure previously described (10) to recover images that are, in principle at least, distortion-free. To illustrate this process we modelled combined dynamic and static distortion, with static distortion as shown in Fig 7 (black). This is a destructive example because all static distortion coincides in direction with that of the dynamic distortion (the direction of the “slow” scan axis). Each frame was also warped dynamically, with distortion in both x and y directions generated from Equation 5 with frequency and phase parameters drawn from the full range described above (see Methods section “Simulating distortion”). The distorting function amplitude here was fixed at 5 pixels to ensure dynamic warp of all frames (i.e. contrary to real data, there is no single frame which could match the ground truth). Example frames warped by both static and dynamic distortion are shown in Fig 8 (A, B).”

The referred-to Methods section was given just above.

In regard to Fig 8, we have chosen to retain parameter information in the Results, because a) the parameters concern those relevant to the previously published algorithm (ref. 10) and it would be confusing to list them here; b) there would be information-overload in the Methods if we explained the whole philosophy of combined static and dynamic distortions, involving clusters of many frames, before presenting even basic results regarding static distortions.

The paragraph implicated is as follows, where we have made some small changes that ought to alleviate the reviewer’s concerns:

Line 546: “Distorted frames were generated in 6 “clusters”, with each cluster having slightly different mean gaze position (up to 10 pixels) as required by the DIOS algorithm. Each cluster also had a different torsional component (up to 10°), to demonstrate robustness to image rotations. Clusters consisted of 1000 frames to ensure that bias resulting from dynamic warp would trend close to zero. Unlike the simulation of random static distortions that was shown in Fig 6, there was no special requirement imposed on gaze position within a cluster, other than that it be randomly drawn from a normal distribution (scaled so that max. amplitude within a cluster was 50 pixels). The simulated eye movement pattern within each cluster was independent from the other clusters. The eye position for all frames and all clusters is indicated in Fig 8C (coloured dots; a different colour for each cluster), overlaid on the ground truth image.”

In addition to the above, we have omitted the information concerning array size for the simulations from the text introducing each Results figure, as this was already specified in the Methods section (“Array size”) and may have contributed to the confusion. The listed array size of 200x200 will be understood to apply to all simulations.

*--Systematic and dynamic distortion*

*As shown in Figs. 7 and 8, and discussed by the authors, there will be the interaction between distortion and eye movement. The authors conclude that the combination of the presented method (DIOS) and the previous method (dynamic recovery procedure) correct the distortions well.*

*This is a surprising result because the principle of DIOS assumes a common distortion (f) among image pairs. Did the authors apply common within-cluster eye movements for all clusters? Why is only one image (Fig. 8F) presented? What about other 5 images after the DIOS procedure?*

Only one image is presented because the dimensionality of the figure would be awkward otherwise. Other recovered images look practically identical. We can assure the reviewer there is nothing untoward happening here. The “special ingredient” which transforms this result from surprising to a “fait accompli” is that there are a large number of frames in each cluster. We have added the following point to the Discussion, as we agree that it is worthy of further comment:

Line 632: “It may at first appear surprising that the output of the previously described dynamic correction procedure would combine well with the newly proposed static correction procedure. However, this marriage is assured by the large number of frames that we included within each cluster. This means that the interaction occurring between systematic and dynamic warp within one cluster should be expected to be nearly identical to any such interaction taking place in another cluster. In other words, the residual static error should not differ appreciably between clusters, given the large number of frames within each cluster. This satisfies the conditions for successful application of the DIOS procedure (a distortion function that is common to all clusters), such that near-perfect correction should be expected. It was noteworthy that although the residual static correction obtained was not simply equal to the underlying static correction applied.”

*If the authors' insight is correct, the discrepancy of the solution from the set systematic distortion is coming from the interaction between the distortion and eye movements. Then, the solution for correcting distortions depends on eye movement.*

*In order to support their conclusions, the authors need to simulate independent random intra-cluster eye movements and compare the similarity between the final corrected images with DIOS derived from each cluster.*

The eye movements were indeed generated randomly (i.e. independently, from a different seed to the random number generator) in each cluster. There was not, as implied in the previous comment, a “common within-cluster eye movement for all clusters”.

We have clarified this with a small addition to the description of cluster parameters that was given above, reproduced here for convenience:

Line 546: “Distorted frames were generated in 6 “clusters”, with each cluster having slightly different mean position (up to 10 pixels) as required by the DIOS algorithm. Each cluster also had a different torsional component (up to 10°), to demonstrate robustness to image rotations. Clusters consisted of 1000 frames to ensure that bias resulting from dynamic warp would trend close to zero. Unlike the simulation of random static distortions that was shown in Fig 6, there was no special requirement imposed on gaze position within a cluster, other than that it be randomly drawn from a normal distribution (scaled so that max. amplitude within a cluster was 50 pixels). The eye movement pattern within each cluster was independent from the other clusters. The eye position for all frames and all clusters is indicated in Fig 8C (coloured dots; a different colour for each cluster), overlaid on the ground truth image.”

In regards to the latter point that we should compare the similarity of the recovered images – they are essentially identical, as expected because

a) DIOS solves for the distorting function that is common to the set of input images
b) Each input image was derived from a cluster of a very large number of dynamically warped images, essentially allowing the dynamic warp to be neutralised

We have added the following to the Results:

Line 564: “…This can be further verified by the similarity with the ground truth image of Figure 8C (correlation = 0.991; recovering the images drawn from other clusters gave near-identical results) and by the reference constellation (overlap of yellow and red polygons in Figure 8F).”

**Reviewer #2:**

*This paper describes a method for correction of static distortion in an imaging system. This was a surprise to me, after having read only the title and the first sentence of the abstract, which led me to believe that the paper would treat the dynamic distortion caused by the interaction of intra-frame motion of the eye with a scanning system. In fact, this paper does not address that particular problem at all. For that reason, I would retitle the paper, and rewrite the abstract, possibly eliminating mention of dynamic distortion in the abstract, but in any case not leading with it!*

This is a fair comment – we have altered the title and the abstract as follows.

TITLE: “Towards distortion-free imaging of the eye”

ABSTRACT: The high power of the eye and optical components used to image it result in “static” distortion, remaining constant across acquired retinal images. In addition, raster-based systems sample points or lines of the image over time, suffering from “dynamic” distortion due to the constant motion of the eye. We recently described an algorithm which corrects for the latter problem but is entirely blind to the former…”

*It is really not clear to me that the method as presented has anything to do with the eye or ocular imaging at all. Clearly the authors hope to apply it to that domain, and have used high-magnification retinal images as their test images in their simulations. But the synthetic distortions that are corrected are arbitrary sinusoidal warps, that to my knowledge are common distortions in any sort of imaging system, ocular or otherwise.*

This is true. We do believe that the method has wide applicability but were a little hesitant to reach outside our field of expertise. Emboldened by the reviewer’s comment, we have made the following additions:

ABSTRACT: “…The method could be extended beyond ocular imaging, to any kind of imaging system in which the image can move or be made to move across the detector.”

Line 691 (Discussion): “…It should be noted that the general nature of our simulations suggest that the proposed method could be applied to imaging systems of arbitrary type, not just the eye. As long as it is possible to perform diffeomorphic registration between acquired images, the method should be applicable to any imaging system which suffers from static distortion and where it is possible (or, as in the eye, unavoidable) to pan the scene obliquely across the image array.”

*Having reread the abstract, it is not obvious that I have fully understood the paper, as the sentence "the method successfully handles torsional movement of the eye" seems to go beyond correction of a static distortion. But I will leave that aside for the moment, and comment on the paper as I currently understand it.*

This comment was added for our colleagues in the ophthalmic imaging community who are concerned with current methods to register images from raster-based devices using “strip” methods that may not perform well when the eye rotates. We have expanded on this a little with the following addition to the Methods, section “Affine transformation of co-ordinates”

Line 304: “It will be shown below that using the above transformations, rotation of the scene does not present any difficulty for the proposed algorithm. However, rotations must be estimated accurately for input to the algorithm; this can be a challenging task depending on the particular imaging modality (35, 36). Image registration is detailed in the next section.”

References 35 and 36 are new to the paper, supporting this point.

*In the third paragraph of the introduction, the authors mention several factors that can lead to "systematic distortion" (which I referred to above as "static distortion"). What is the magnitude of these problems in actual instruments? Were the authors led to this problem because systematic distortion is a problem in their own instrument?*

This is a difficult problem to answer, because of the lack of experimental data. We have added several germane points to the Introduction:

Line 55: “To our knowledge the problem of correcting for static distortion does not appear to have been canvased in the ophthalmic imaging literature. It is difficult to state precisely how significant a problem this is, due to a lack of experimental data available on distortion of the eye (21), let alone the combination of eye and imaging system. For the eye alone, schematic eye models can be used to provide some estimate. Over a typical field of view of ~30° in a conventional ophthalmoscope pointed at the optic nerve head (15° off-axis), distortion from one edge of the field to the other is predicted at ~6% (21). This is 1.8°, approximately one third of the diameter of the optic nerve head. Distortion is likely to be even higher a) when including the optics of the imaging system, which lack the excellent wide-field imaging properties afforded by the gradient index of the crystalline lens (22); b) with an adaptive optics correction in place, as off-axis aberrations typically accrue more rapidly across the field than without adaptive optics (23); and c) when constructing montages of the retina or imaging with very wide field devices (24), which can span >180°.

As opposed to the field of ophthalmic imaging, there has been extensive work on the problem of static distortion in the field of… “

Interestingly, our method can be used to answer the question of just how much distortion is present. We have added the following to the Discussion:

Line 582: “We have demonstrated a new method to estimate and correct for static distortions in an imaging system”

Line 665: “For retinal imaging systems adopting a wider field of view than a typical adaptive optics design, distortion is likely to be far greater as discussed in the Introduction. As noted in that section, there is a lack of experimental evidence regarding the degree of distortion imposed by the eye in conjunction with typical imaging systems. However, our method can be used to redress this lack of evidence, being applicable to data acquired from any ophthalmic imaging device. If the degree of distortion calculated is found to be substantial, the proposed algorithm can then be implemented in the image processing software of the particular device to correct image data saved in the future, and can also be applied post hoc to correct previously acquired data if multiple frames were recorded.”

Finally, we note further that the genesis of this algorithm was in the adaptive optics psychophysics workshop (AOPW) convened by Prof Austin Roorda of UC Berkeley, held online in June 2020. During the workshop, our prior algorithm regarding correction of dynamic distortion was discussed. Researchers from several groups lamented that the method would be blind to any systematic distortion present which, in particular, frustrates attempts to create wide-field montages of the retina. This inspired us to develop the DIOS procedure. Given this genesis, we can certainly answer in the affirmative that static distortion is perceived to be a limiting factor in the design of AO retinal imaging systems and in the analysis of data captured by them.

The answers just given to this comment also serve to address a comment made by the reviewer below, regarding the desire to see a “more realistic” set of synthetic images. Because distortion is indeed a problem in AO imaging, we do regard the use of distorted AO images as a sufficiently realistic exercise.

*The authors liken their problem to calibration of the intrinsic camera parameters in computer vision, and refer to "self calibration," with a number of relatively recent references given. I would like to draw the authors' attention to a 1989 paper by Maloney & Ahumada (Maloney, L., & Ahumada, A. J. (1989). Learning by assertion: A method for calibrating a simple visual system. Neural Computation, 1, 387-395) that addresses a related problem, namely how the brain "calibrates" itself to compensate for disarray in the positions of the photoreceptors. The represents an extreme form of systematic distortion, in which each input pixel is displaced in the imaging plane by a random amount. Their solution works by imposing a condition they call "translation invariance," which states that the image of a translated scene should be an equivalent translation of the image of the original scene, or I( T(s,delta) ) = T( I(s), delta ). If I have understood the present paper correctly, the method describe employs the same principle. I think the fact that I am not 100% sure means that the exposition could be clarified somewhat; I suggest starting out with a clear statement of the principle behind the solution (whether it be translation invariance or something else), before delving into the details. The original Maloney & Ahumada work assumed the motion generating the translations was known exactly (perhaps using an efference copy of the eye movement signals, or proprioception); later work explored the performance of the method using imperfectly known motion estimated from the raw uncorrected images themselves, which seems analgous to the problem of calibrating an ocular imaging system.*

Thank you for pointing us to this reference, which we were not previously aware of. It is a very interesting idea. There are indeed strong philosophical similarities; we have added the following to the Introduction:

Line 103: “A form of non-parametric self-calibration has also been proposed in the field of vision science, not for imaging per se but for a visual system to compensate for distortion imposed by the optics of the eye and by irregular distribution of photoreceptor cells tiling the retina (33). In that work it was recognised that movements of the eye shift the retinal scene and that, if the objects in the scene are actually stationary and the system well calibrated, all objects in the scene should move by the same amount. If the calibration is in error, this will no longer hold true. Therefore trial-and-error may be used to modify the system’s calibration function until the distortion-on-movement is minimised. This is a philosophically similar approach to the one described below, despite the different application. However, major limitations include the difficulty of ensuring convergence of the solution on a global minimum, the potentially large number of image samples required, and a “lowpass” assumption of image formation; these limitations are avoided the present work.”

Given the similarity in thinking between these approaches, we think it appropriate to discuss the application of the new algorithm beyond image processing, in calibrating a visual system. We have added the following to the Discussion:

“Finally, the method advanced here has application beyond simple imaging, to understanding the evolution of visual systems and machine vision. It is intriguing to wonder whether the small, incessant movements made by the fixating eye may have evolved, in part, due to their utility in calibrating the visual scene. This would be a useful ability for an eye to develop due to its high optical power and non-regular arrangement of receptor elements. Previous work has described how comparison of the visual scene after small movements of the eye/head might be used to iteratively arrive at a satisfactory calibration for such causes of distortion (33). The present work extends such thinking, providing an analytical solution that can be solved with only a handful of samples of the visual scene. We have also shown that the method can be augmented to handle temporal variations, which could arise due to non-synchronous firing of neurons or due to random movement within the scene, as occurs for example by shimmer arising from the ground on a hot day.”

*I did not find the one-dimensional example particularly helpful. Writing the equations in vector form can make them as concise as a 1-D solution, but then you would be done.*

The problem cannot be vectorized *per* se, although the distortion maps are represented by column vectors. At any rate, Equation 2 could not be made any more concise. In regards to the section containing the 1D example, it sounds as though the reviewer is suggesting that we remove the section. We would be loathe to do this, as it provides close to the simplest possible example, which will assist readers with less mathematical acumen than the reviewer (probably most readers!) to appreciate the truth of Equation 2 and hence to understand how the algorithm works.

*Here is what I would ideally like to see: apply the method to a more realistic set of synthetic images, generated by taking a large retinal image as ground truth, and then simulating capture through an imaging system containing realistic distortion as might be encountered in practice. First do it for a flood-illuminated system, so that dynamic distortions are not an issue; then simulate dynamic distortions in a scanned system by simulating sampling the image sequentially in time while it is moved according to a realistic eye movement signal (with and without torsion).*

We do agree that this sounds like a sensible series of steps, and it coincides in large part with the systematic approach undertaken in the paper. It is possible that there is some confusion on the part of the reviewer, who later notes “*Line 55: “To our knowledge none of the methods advance have treated the case of dynamic and systematic distortions together…” – this certainly leads one to expect that the authors will treat them together, but that does not seem to be the case.”* Both dynamic and static distortion are certainly compared, as should be clear from the abstract, from the Results section titled “Static and dynamic distortion”, and from the title of Fig 8 “Correction of simultaneous static and dynamic distortion.”

At any rate, we provide commentary and updates to the paper based on each of the points raised, in turn below:

- *apply the method to a more realistic set of synthetic images generated by taking a large retinal image as ground truth*

Thank you for this suggestion, please see the modifications made above in answer to these earlier questions from the reviewer: “W*hat is the magnitude of these problems in actual instruments? Were the authors led to this problem because systematic distortion is a problem in their own instrument?”* This material should now make the case convincing that distortion is indeed a problem for AO imaging, and hence the use of our distorted AO images is a sufficiently realistic exercise. As given above, the changes made begin in paragraphs starting on Lines 55 and 665.

It should further be noted that the type of images used does not really matter, other than that cone photoreceptor images do provide a particularly useful image to appreciate subtle differences in distortion across the field. This argument is made with the following addition to the section “Ground Truth” Data:

Line 357: “It should be noted that the algorithm described here operates on image registration data, rather than on the images themselves. As described above (see section “Image Registration”), for the majority of data presented here we carried out idealised registration does not consider the content of the images themselves; the images are merely useful tools to illustrate the degree of distortion. Hence, the use of cone photoreceptor images does not diminish the applicability of the proposed method to other types of images. The uniform, high spatial frequency content of such images does provide an excellent canvas in which to make comparisons, compared for example to images collected by other modalities which may lack “interesting” information across broad regions of the image, potentially masking residual distortion. These characteristics of the images used also lend themselves well to application of widely available techniques for diffeomorphic registration, as applied in Supporting Information 1. ”

- *and then simulating capture through an imaging system containing realistic distortion as might be encountered in practice*

This comment is at odds with the reviewer’s comment above, with which we agree, that “*the synthetic distortions that are corrected are arbitrary sinusoidal warps, that to my knowledge are common distortions in any sort of imaging system*”.

However, again in response to the questions “W*hat is the magnitude of these problems in actual instruments? Were the authors led to this problem because systematic distortion is a problem in their own instrument?”*, we noted that there is a lack of experimental evidence regarding the degree of distortion expected in real eyes, especially in conjunction with an imaging system which is likely to significantly contribute to distortion. Again, please see changes made in paragraphs beginning on Lines 55 and 665.

Rather than base our modelling on such dubious estimates as are available in the literature, our strategy was to make sure that we modelled distortion that is much worse than likely to be encountered in practice (example Fig 6A). This is encapsulated by the following addition at the beginning of the section, “Simulating distortion”

Line 386: “As described in the Introduction, it is difficult to know how much and what sort of static distortion is present in ophthalmic imaging systems. Our general philosophy was therefore to simulate distortion of greater magnitude and with more complex structure across the field than we believe might be encountered in practice. This should serve to convince the reader of the robustness of the proposed method.”

Line 400: “The above parameters allowed us to test distortions of high amplitude, and with unusual structure (e.g. asymmetric, minimum not coinciding with the array centre, multiple cycles across the image) to ensure robustness of our approach.”

- *First do it for a flood-illuminated system, so that dynamic distortions are not an issue;*

This is already done (we do not think the reviewer was making a point-by-point list of changes, but rather illustrating on the whole what they would do, which has some overlap with what was already done).

- *then simulate dynamic distortions in a scanned system by simulating sampling the image sequentially in time while it is moved*

This is precisely what was done, however…

- *according to a realistic eye movement signal*

… we purposefully did not use such a signal, although this is the approach that we previously undertook (Ref 10). Some members of the community (in private discussions) have proposed that the pattern of eye movements shown previously may simply have been fortuitous for the purposes of image recovery. We therefore undertook to confront our algorithms with a more difficult scenario as described below, now with additional supporting arguments following the reviewer’s comment:

Line 413: “Although the sinusoidal distortions modelled do not have particular “real life” analogues (the eye is unlikely to follow sinusoidal motion), this approach allowed simulation of distorting functions that varied widely in degree and spatial structure in an unbiased manner. There is also no correlation in distortion applied between one frame and the next as would occur for a real eye. This helps to avoid a potential criticism of our previous work (10), where it might be supposed that the eye movement data underlying our simulations happened to be a fortuitous set for the purposes of image recovery. We note further that our previous work (10) and its subsequent application by others (12-17) have already confirmed the previous method to correct for dynamic distortion in the presence of realistic eye movement patterns. In the modelling below, we will show that as long as a satisfactory dynamic correction is obtained, the new static correction procedure can then be applied to recover images that are free of both kinds of distortion. The significant distortion entailed by the present modelling can be appreciated by the sequence of 100 consecutive frames shown in Supporting Information 2.”

To provide further assurance that we have indeed considered substantial and realistic-appearing intraframe distortion, we have also now included Supporting Information 2 as cited in the addition above. This is an image stack which the reader can animate as a short video to show the large dynamic distortion seen from frame to frame. We believe there will be little doubt for most readers that we have not challenged our algorithm sufficiently.

- *(with and without torsion).*

Whilst it is sensible to undertake the exercise with and without torsion, it is a more difficult problem in the presence of torsion. Given that we have obtained excellent results with torsion, there is little further benefit to present the reader with the torsion-free results.

*It is hard to know how to interpret the results shown in figure 7; can error bounds be put on the solution? Some statistics might help here.*

This is a reasonable comment. There are no applicable error bounds, but we have added RMS error estimates to the text. These verify the very small differences between the applicable curves (RMS = 0.17 – 0.19 pixels), and support the already given figures regarding correlation between the recovered images. The new numbers are given on lines 566, 576 and 582.

*Minor points:*

*line 50: I would have thought that in the computer vision context, "rotation" of the camera includes pan and tilt, which for small angles translates the image, but is properly a rotation. To use this for camera calibration, it is necessary for the axis of rotation to pass through the optical nodal point, which requires some care.*

Whilst the term “rotation” in common usage has some ambiguity, there is no ambiguity in the reference cited. The author refers to rotation and translation of co-ordinate systems, which matches our usage here.

*Line 55: “To our knowledge none of the methods advance have treated the case of dynamic and systematic distortions together…” – this certainly leads one to expect that the authors will treat them together, but that does not seem to be the case.*

As mentioned above, we are a little dismayed that the reviewer could be under the impression that we did not treat static and dynamic distortion together, as should be clear from (among other places) the Abstract, from the Results section titled “Static and dynamic distortion”, and from the title of Fig 8 “Correction of simultaneous static and dynamic distortion.”

*line 65: It seems the word "not" may be missing?*

*line 79: "... such any one of the ... images " is the word "that" missing after "such"?*

Thank you for picking up these oversights, which we have now corrected.

*Table 1: The final row appears to be a map from the first "image" to the second, but the legend states the opposite.*

The mapping given allows image 2 to be converted into image 1; it provides the location of the intensity information to be placed at the given pixel. Because object “H” is two units to the left, the value recorded is -2.

*line 190: A little more explanation of the mathematics might be appropriate here. Matrix A is known and is sparse, but has dimensions N x M*N... Apparently the inverse matrix is really a pseudo-inverse? The description of lsqminnorm sounds like it is applied to under-constrained problems... But this problem is not under-constrained?*

We have now clarified as follows:

“The array A is very large. If N is the number of pixels on the detector and M the number of image pairs considered (several images being recommended to ensure the solution is adequately constrained), there will be M*N observations (M*N rows) of the N variables (N columns). A is therefore a pseudoinverse. For most modern images A will be a very large array whose (pseudo)inverse would be prohibitively resource intensive to compute. However, it is not necessary to compute the inverse directly. Since the matrix is sparse there exists an efficient solution for the expression A-1 Δ through minimization of least squares. We implemented this using the Matlab function “lsqminnorm” (MATLAB R2017b, The Mathworks, USA). Although this function has additional benefits in selecting an appropriate solution to under-constrained problems, we found that it was necessary to constrain the problem by other means as detailed below.”

*line 258: perfect registration is assumed; How robust is the method to registration errors - isn't that important?*

As described in this section (“Image Registration”), it is important to differentiate the performance of the algorithm itself from the performance of image registration approaches, which are famously prone to error.

However, to assist readers considering adoption of this algorithm with the help of our code supplied in Supporting Information 1, we have now added an extra parameter to inject noise of adjustable amplitude into the generated image stack. This results in inaccurate image registration whose influence on the recovered image quality can now be readily appreciated. Readers are now pointed to this functionality by the following:

Line 325: “… The robustness of the proposed algorithm to errors in image registration may be explored by increasing the noise parameter included in Supporting Information 1.”

*Equation 6: Why is Dx only a function of x, and Dy only a function of y? (compression, but no shear) This appears not to be the case at line 324, where Dx(y) and Dy(y) are referenced?*

The method does not have any problem with Dx and Dy varying in both x and y. But it does make the results easier to follow (for example, by allowing us to plot the mean recovered distortion profile). We have now stipulated this as follows:

Line 398: “… It should be noted that there is no requirement that Dx, for example, varies with x but not with y. This does, however, make it easier to visualize the results because the distortion map can be meaningfully plotted in one dimension.”

In regards to the case at line 324 in the original submission, this was an error in the equation (now Equation 5) that we have now corrected: Dx and Dy are both functions of x and y (i.e. are 2D functions), but only vary in one dimension or the other.

*Line 459: The polygons are not that easy to compare visually - is it not possible to come up with a quantitative measure?*

We now quote an example correlation between 8D and the ground truth of 0.67 (line 562), which compares favourably with the figure of 0.99 given later in the paragraph after the DIOS procedure). The polygons do not lend themselves particularly to quantification, but are meant to provide a visual guide in the context of a static figure, where it can be hard to compare changes.

*Line 498: Why can’t the magnitude of the distortion be solved? Is this just saying that there is an unrecoverable constant rigid transformation (like a prismatic shift)?*

Yes, precisely. We have clarified as follows:

Line 599: “…The method is blind to the absolute value of the distorting function (for example, a constant shift applied to all images), however, such constant shifts merely move the image rather than producing distortion.”

*Line 543: Instead of referring to data in figure 7 by color, state what the different colored curves represent.*

We have clarified as follows:

Line 656: “…(e.g. the difference between red/magenta and the other curves in Figure 7, showing respectively that the underlying static distortion is not necessarily equal to the apparent or required static distortion).”

*Line 546: It is assumed that eye movements are negligible compared to the speed of the fast scan in an SLO. This may not be true for saccadic velocities.*

This is true. We have added the following to the Methods section, “Simulating distortion”

Line 411: “…This assumption could be violated during very fast (saccadic) eye movements, however, we do not consider such movements here due to the irrecoverable motion blur which would result.”
