# Supplementary material for: Towards distortion-free imaging of the eye
Source: PLoS One. 2021 Jun 10;16(6):e0252876. doi: 10.1371/journal.pone.0252876 (PMC8192003; doi:10.1371/journal.pone.0252876)
Supplement: Attachment — Submitted filename: Response to Reviewers.docx [file pone.0252876.s004.docx]

We thank the editor and both reviewers again for their careful consideration of this manuscript. We address the remaining reviewer comments below. Please find pertinent comments reproduced in *italics*, with our replies following in plain text and passages from the manuscript reproduced in “quotations” with new additions marked in red. Line numbers refer to the “tracked changes” version of the revised manuscript, with “All Markup” visible.

**Editor**

*While new concerns were raised in this round of review, particularly by reviewer 2, i.e. eye position related distortion, that may help further improve the simulation, you do not have to agree to all the points. A balanced discussion of the potential limitation of the simulation work may help address the review concern.*

We thank the editor for this suggestion. We have endeavoured to follow the reviewer’s suggestions where practical below, or to at least add discussion of each point to the manuscript.

**Reviewer #1**

*The authors extensively addressed the reviewers' concerns and the manuscript is well revised.*

*I only have a few concerns.*

*- Eye motion simulation*

*According to the details of the description of the presented simulation, the authors assume that the eye motion (full-field movements + dynamic distortion) has no variation in the horizontal component during single image acquisition (Dx(y) is a constant).*

*Even if there is a sinusoidal horizontal dynamic distortion, it is not a realistic simulation, but I think it is better to remind readers of this point.*

We have made this point more explicit as follows: (Line 414)

“There were accordingly 6 parameters set for each distorting function. It should be noted that the horizontal distortion in Equation 5 varies only in x, and the vertical distortion only in y. This makes it easier to visualize the results because the distortion map can be meaningfully plotted in one dimension. However the method is not limited to application in this way and works just as well for arbitrary distortions. Similarly, the method works just as well for simpler distortions, for example the rotationally symmetric distortions expected for a centred, high power system. We chose to present results from sinusoidal distortions here, in part because they look sufficiently bizarre to convince the reader of the broad applicability of the method, and in part because of the well-known Fourier decomposition theorem which allows arbitrary shapes to be reconstructed from a sinusoidal series.”

*- Comparison of 6-cluster outputs with a ground truth*

*The authors described the similarity between the outputs and a ground truth that "This can be further verified by the similarity with the ground truth image of Fig 8C (correlation = 0.991; recovering the images drawn from other clusters gave near-identical results) and ..."*

*I suggest describing the range of correlation coefficient to support their claim.*

We have now added the following (Line 593):

“This can be further verified by the similarity with the ground truth image of Fig 8C (correlation = 0.991; recovering the images drawn from other clusters gave near-identical results with the worst correlation at 0.986) and…”

*- Fig 8*

*"... running on the six outputs of dynamic recovery (one for each cluster), examples of which were shown in B and D."*

*Aren't they "D and E"?*

Yes, and thank you. This has now been fixed (Line 569):

“dynamic recovery (one for each cluster), examples of which were shown in D and E.”

**Reviewer #2**

*In general, the authors have made a good effort to respond to the concerns raised by the original reviews.*

*I have one serious concern that did not occur to me when I read the paper for the first time a few months ago: the method explicitly solves for "static" distortion that is the same on every frame. It does this by comparing the positions of features in pairs of retinal images, obtained with the eye in a different pose. The method must assume that either the stationary eye does not contribute any distortion itself, or that whatever distortion it does contribute is constant (does not depend on eye position). I am doubtful that either one of these assumptions hold; in fact, the authors themselves mention distortion contributed by the eye at line 57. This really needs to be addressed.*

This comment shows that we have failed to fully describe the sources of static distortion in ophthalmic imagery. We had mentioned some of the following in the Abstract but not in the Introduction. This oversight has now been remedied (Line 48):

“We refer to this as static distortion. Such distortion will occur in any ophthalmic imaging system due to marked anisoplanatism which results from the high power of the eye and of the optical systems used to image it, which is one reason that adaptive optics systems are typically limited to 1-2° in diameter (18, 19). Major distortion also arises when imaging over an extended field, due to the typically flat shape of detectors compared with the highly curved retina.”

Reference 19 had already been cited elsewhere in the manuscript, but is relevant here as one of the few works to discuss limitations to isoplanatism imposed by the optics of imaging systems as opposed to just the eye.

The addition of this statement should make it clear that we advocate the second assumption proposed by the reviewer above, that “*whatever distortion [the stationary eye] does contribute is constant (does not depend on eye position)*”. This assumption is justified based on the very small amplitude of eye movements required for the new method, relative to the size of the isoplanatic region of the eye, as now made more explicit below: (Line 134)

“Solution of this sparse system of linear equations gives the common warp field that was encountered by each frame, as detailed further below. This approach implicitly assumes that the distortion conferred by the eye itself does not change to any appreciable degree over the range of eye positions considered. This assumption is justified given that the approach requires displacements of only a few pixels (each one corresponding to ~ 0.5 µm in our images), compared with the extent of the isoplanatic zone of the eye which is typically in excess of 300 µm (18).”

*I am trying to imagine what would happen when a distortion-free imaging system is used to acquire retinal imagery from an eye exhibiting position-dependent distortion. I haven't been able to completely wrap my head around it, but I am confident that the result will not be an undistorted image of the retina, because the assumptions of the algorithm are violated.*

The reviewer is correct, but the effect is a matter of degree. The displacements required for the algorithm are two orders of magnitude below those required to produce a measurable change in wavefront aberration of the eye. This should now be clear from the above addition.

*In practice, one approach to this problem could be to try to calibrate the instrument without an eye, by placing a calibration target at the focal plane of the device and moving it. Then when trying to estimate the (possibly position-dependent) distortion added by the eye, the instrument distortion could be factored out.*

This is a complexity that the present algorithm seeks to avoid – now justified given the extra material added above re: the isoplanatic zone of the eye.

*Before moving to my comments on the revised manuscript, I would like to respond to a few of the responses to my original review:*

*"The problem cannot be vectorized per se, although the distortion maps are represented by column vectors. At any rate, Eq. 2 could not be made any more concise."*

*I certainly agree with the second statement, Eq. 2 is great as it stands, and should be equally true for the two-dimensional problem of interest. One equation that I would like to see in vector form is Eq. 3, which is written out as two equations, one for the x coordinate and another for the y coordinate. The x and y coordinates could be subsumed into a position p, and equation 3 could then be written in a coordinate-free way as:*

*f(p_R) - f(p_M) = p_M - R p_M - T*

*where R and T are the rotational and translational components of the affine transformation. This form still requires the definition of an origin, i.e. the location of the zero-length position vector that is the center of the rotation. As far as I can tell, this equation should be the same as equation 1. I really think that it the method can be described a little more clearly then the one-dimensional example will not really add anything.*

Whilst we agree that this would be more economical, if the reader would like to implement the algorithm they will have to translate from this mode of presentation into the form that we originally offered. We believe it more instructive to give the explicit equations required.

We have added the following text to alert the reader to the economy suggested by the reviewer: (Line 314)

“Hence both the equations in x and in y have terms which account for the effect of rotations and translations.”

*Further on, it appears that there was a typo in my original review, as evidenced by the authors' response, "This comment is ad odds with the reviewer's comment above, with which we agree, that 'the synthetic distortions that are corrected are arbitrary sinusoidal warps, that to my knowledge are common distortions in any sort of imaging system'." What I meant to say in the original review is that to the best of my knowledge these are NOT common distortions in any sort of imaging system. I apologize for my sloppy proof-reading. That being said, I grant that any arbitrary distortion function can be synthesized from sinusoidal warps via Fourier series, so this doesn't seem like a critical issue; as the sythetic distortions appear to be quite large (possibly larger than what might be encountered in practice), if the algorithm can handle them then it will probably work well in practice. Still, it might be nice to test with a circularly-symmetric distortion function, similar to what might be encountered with a lens.*

We have indeed already tested a variety of functions not shown in the paper. To paraphrase the reviewer, if the algorithm works for distorting functions as bizarre as some of the more extreme sinusoidal distortions shown here, it should work for just about anything. Whilst we would prefer not to add another example to the paper in the interests of brevity, we offer the results below for a rotationally symmetric distortion, for the reviewer and editor to verify the veracity of this claim. Note the scale for the bottom row, which shows error in estimation of the distorting function is on the order of 1/1000^th^ of a pixel.

In response to a comment above from the first reviewer, we had added the following prose which serves to encapsulate our reasoning for including sinusoidal distortions and also incorporates the reviewer’s point regarding Fourier series. Hopefully the figure inserted above for the editor/reviewer will convince of the truth of this claim despite it not being shown in the paper.

(Line 418)

“…Similarly, the method works just as well for simpler distortions, for example the rotationally symmetric distortions expected for a centred, high power system. We chose to present results from sinusoidal distortions here, in part because they look sufficiently bizarre to convince the reader of the broad applicability of the method, and in part because of the well-known Fourier decomposition theorem which allows arbitrary shapes to be reconstructed from a sinusoidal series.”

*In their response to another point, the authors state that, "there is a lack of experimental evidence regarding the degree of distortion expected in real eyes." I would like to note that there is a relationship between geometric distortion and optical aberrations, and there are databases of wave aberration functions measured on collections of eyes, from which it might be possible to make reasonable estimates of the geometric distortions encountered in real eyes. This illustrates a problem with the approach, however, which is perhaps unique to the problem of ocular imaging: instrument-related distortions can safely be assumed to depend only on the sensor pixel location. Eye-related distortions, on the other hand, maybe unique for each different pose of the eye. This seems to me to be a possibly-fatal flaw with the whole approach, given that the method relies upon eye movements to produce the image shifts, and assumes that the distortion function is constant and locked to the sensor. This problem must be addressed, if only by estimating the eye-related distortions from aberration data, and showing that they are insignificant. It also seems possible that they might become significant for highly-aberrated eyes, and this should be considered also.*

As now added to the manuscript following a comment above, eye-related distortions will certainly be significant, and are indeed part of the problem being addressed by the algorithm along with distortions from the imaging system itself. As also justified above, these distortions do not change appreciably over the small movements of the eye required of the algorithm.

*I have a serious concern with a statement made on line 473: the authors demonstrate that when only two images are input, which are vertically offset, that the horizontal distortion is not recovered with as much accuracy. The authors conclude that this can be fixed by shifting obliquely. This does not seem intuitively correct to me; it should be possible to formulate the problem in a coordinate-free way, in which case I would expect that larger errors would be encountered in the dimension orthogonal to the shift, regardless of what direction that might happen to be. For an oblique shift, the largest errors should be found in the orthogonal oblique direction. This is just my intuition, of course, but it seems highly unlikely to me that the coordinate axes are special, and this seems even more critical when this principle has been immortalized as part of the name of their procedure.*

We appreciate the reviewer’s intuition, and have systematically tested this ourselves. The referred to section of the manuscript introduces Figure 4, which was designed to explicitly demonstrate that no information is gained in a direction in which no displacement has been made (the Figure also demonstrates another limitation, the requirement for more than two image frames). The blue “flatline” of Figure 4 confirms that the algorithm recovered no information regarding horizontal distortion in this example.

The reviewer might be further convinced by considering Equation 1:

$$f\left( x_{R} \right)-f\left( x_{M} \right)= x_{M}-x_{R}-\left( T_{M}-T_{R} \right)$$

The “paired functional difference” in the x direction is learnt by comparing images that have been translated in the x direction. If the eye does not shift in the x direction then (T_M_ – T_R_) is zero, therefore the location of any given feature will not change its x co-ordinate so (x_m_ – x_r_) is also zero. Hence, the “paired functional difference” will be zero. We would essentially be investigating the same portion on the distorting function between frames, so cannot learn the difference *across* the function, which is what the algorithm attempts to do.

The only caveat to this example might be where the horizontal distorting function is a function of vertical displacement, such that different portions on the horizontal distorting function could indeed be encountered in the absence of a horizontal displacement (assuming there is a vertical displacement). However, without prior knowledge of the distorting function this would not be a reliable method to base the algorithm upon.

*Detailed comments:*

*Line 65: It is stated that "Distortion is likely to be even higher ... when constructing montages of the retina or imaging with very wide field devices." In this case, it is not clear to me that the problem is distortion, so much as the fact the the retina is not flat, so any attempt to create a montage by translating and registering flat images is doomed to have errors, even in the absence of distortion. The projection of a spherical surface onto a planar image sensor could produce the appearance of distortion when in fact it is an inherent property of the object being imaged, not the imaging system.*

As a counterpoint, consider that any optical distortion could be dealt with by shaping the detector in an appropriate fashion. It then becomes difficult to decide what is “optical” and what is “retinal” distortion. Whether one attributes the “fault” to the retina for not being flat like the sensor, or to the sensor for not being curved like the retina, is moot to the algorithm. We have now properly introduced the various sources of distortion in the Introduction in response to a comment above. We reproduce the change here for convenience: (Line 48)

“We refer to this as static distortion. Such distortion will occur in any ophthalmic imaging system due to marked anisoplanatism which results from the high power of the eye and of the optical systems used to image it, which is one reason that adaptive optics systems are typically limited to 1-2° in diameter (18, 19). Major distortion also arises when imaging over an extended field, due to the typically flat shape of detectors compared with the highly curved retina.”

*Line 108: "... trial-and-error may be used to modify the system's calibration function ..." - Note that in reference 33 Maloney & Ahumada do not use "trial-and-error," but rather the Widrow-Hoff learning rule, similar to back-propagation as used in neural networks.*

We have now qualified our description of this as “principled” trial-and-error (Line 110). We would argue that the method does use trial and error, because it consists of a series of iterations in which a solution is trialled, the outcome evaluated, an error statistic computed, and then a new solution trialled. It is not a “blind” trial and error as was implied by our initial description. We do not wish to further describe the nuances of the learning rule because it is not needed in order to understand the paper.

*Line 148: The text says that the letter "H" is highlighted in red, but it does not appear so in my review copy. Consider depicting this in a way which will still work when printed in black-and-white.*

This was left over from the previous version of the manuscript. We have now updated to describe the letter as bolded, rather than red (Line 158).

*Line 159: Xm is referred to as the “moving image”, but wouldn’t it be more accurate to call it the “translated image” or “moved image”?*

We have updated all instances of “moving” to “moved” images.

*Line 160: Even though it is somewhat clear from context, it would be good to define the symbols used in these equations. It is kind of confusing, because it is stated (line 165) that the expressions that are equated to x_0 "refer to 'world' co-ordinates", but clearly x_R and x_M refer to sensor co-ordinates. It would be better not to use the same symbol (x) to refer to values in different domains.*

We have changed this to the symbol x_U_, describing it as the undistorted co-ordinated space rather than “world” co-ordinates: (Line 167)

“Returning to the task of determining the distortion map, the co-ordinates in undistorted space (x_U_) of a given feature can be expressed independently in the co-ordinate system of the reference image (x_R_) and in the co-ordinate system of the image to be registered (the moved image, x_M_):

Reference image:	$\boldsymbol{x}_{\boldsymbol{U}}=\boldsymbol{x}_{\boldsymbol{R}}+f(\boldsymbol{x}_{\boldsymbol{R}})- t_{R}$

Moved image:	$\boldsymbol{x}_{\boldsymbol{U}}=x_{M}+f(\boldsymbol{x}_{\boldsymbol{M}})- t_{M}$

In this particular example (i.e. for the feature H), these expressions evaluate as follows:

Reference image:	$\boldsymbol{x}_{\boldsymbol{U}}=2+1- 0= +3$

Moved image:	$\boldsymbol{x}_{\boldsymbol{U}}=0+0-(-3)= +3$

Given the equality of these expressions (i.e. that they both refer to the undistorted co-ordinate space) we can write…”

*Line 168: The symbol D_M,R is said to represent "the output of pixelwise registration between the two images," and is therefore a vector (with dimensionality equal to the number of pixels), while T_M,R is "the full-field translation of the moving image relative to the reference image," which in this example is a scalar or 1-D vector. In the case of 2-dimensional images, if T is allowed to be an arbitrary rigid transformation (e.g. translation + torsional rotation), the T would need to be different for every pixel and would have the same dimensionality as D. This should be clarified. The authors rejected my suggestion to present the equations in vector form, but I still encourage them to adopt type-setting conventions that help indicate the types of various entities, such as bold non-italic lower-case letters for vectors. Perhaps in this case D and T are really functions of x? This needs to be fixed up so that a reader could easily implement the algorithm themselves from the description.*

We have made the following changes to clarify the dimensionality associated with each of the variables in the equations, as suggested:

- Bolded the vectors and converted them to lower case. This includes the results of image registration D, which is now “**d**”, as well as the co-ordinates in each of the reference and moved image spaces x, which are now “**x”**
- T is a scalar, so was converted to a lower case “t”
- In the text, specifically referred to the above as vectors or scalars as appropriate

These changes can be found in Equation 1 and the text leading up to it, and generally throughout the section titled “Simplified 1D example”. We have also re-done Figure 1 and Figure 2 and their captions to follow these conventions.

We have also removed bolding in Equation 2 and the accompanying text from the non-vector (array) terms to avoid confusion; these are now unbolded capital letters.

*Line 193: The corresponding pixels are denoted x_1 and x_2, while earlier the were x_R and x_M. These should be consistent.*

This comment refers to the caption for Figure 1, where we have numbered pixels on the detector. This figure is used to make the point that in one image (e.g. the Reference) we are sampling the distortion function at one particular pixel (e.g. x2) whilst in the other (e.g. the Moving) we are sampling the distortion function at another pixel (e.g. x0). These are meant to assist in interpretation of Figure 2, where the array A is populated with a different numbered pixel for each column.

To improve the clarity of both figures, we have changed the pixel numbering to accord with the example in Table 1. There is now a row which actually corresponds to the example, and which matches the labelling shown in Figure 1. This row is highlighted in the arrays in the new Figure 2.

The new Figure 1 caption is as follows:

“**Fig 1. Illustration of a paired functional difference**. An example static distortion map f(x) is defined over the pixels of the detector (x). Circles represent examples where detector pixels (x_0_ in the moved image, x_2_ in the reference image) were determined, by image registration, to have “seen” the same object in different frames as was depicted in Table 1. The value of the distorting function at the two locations is f(x_0_), f(x_2_). The object in question has shifted between moved and reference frames by an amount (d_M,R_), which is equivalent to the sum of a) the difference in the distorting function f(x_2_) – f(x_0_), and b) the overall translation of the scene t_M,R_. Therefore, pixel-wise registration between shifted versions of the same scene can be used to build up information about the shape of the distorting function that is common to each frame.”

The new Figure 2 caption is as follows:

“**Fig 2. Linear algebra representation required to solve for the static distortion function f(x).** A sparse array (A) populated by entries of ± 1 is used to perform pairwise difference operations between image pixels x_i_ for the distorting function f(x). The resulting “paired functional differences” are given in Δ. This array is equivalent to the s required to register the two images (d_M,R_), after accounting for the scalar whole-field translation between the images t. The unknown values f(x) can then be solved by pseudoinversion of the large, sparse matrix A. The highlighted row corresponds to the example depicted in Table 1 and in Fig 1.

*Line 217: Why are these equations relegated to a figure instead of typeset in-line, as was done for the previous equations?*

We found that we could not reproduce the desired features of this figure as an equation. This is because we wished to label parts of the arrays to improve clarity for the reader. We think this is vital for intelligibility of the paper, especially given that our original submission lacked clarity for the reviewer who appears far more knowledgeable in mathematics than the average reader of the journal.

*Figure 2: There is something that I don't understand about this: x_1, x_2, etc, which label the columns of A, refer to locations on the sensor, while the vector f is the unknown distortion map that we wish to obtain. Why should there only be rows relating successive locations, such as x_1 and x_2, x_2 and x_3, etc? It seems like x_1 could correspond to any of the other x's for an appropriate motion. This doesn't make a lot of sense to me.*

This was an unfortunate by-product of the example pixel relations that we chose. We have now re-drawn Figure 2 so that it is clear that successive rows need not be related.

*Equation 3 (line 292): T represents an affine transformation matrix, but it is written as a function with two arguments. In equation 4, there appear to be 3 parameters (theta, T_x, and T_y). What are the two arguments in equation 3?*

These were not intended to be function arguments, but entries in the array. We have replaced these with subscripts as conventional and clarified as follows: (Line 309)

“Here the term T indicates a standard affine transformation matrix which can implement any combination of translation, rotation, scale and shear. The numbers in subscript indicate the row and column entries in the transformation matrix. For the example of rigid body transformations (translation and rotation alone), the transformation matrix (T) would appear as…”

*Line 330: "Ground truth" images were obtained from a flood-illumination ophthalmoscope, and subsequently distorted with known distortions. While mostly irrelevant for the work presented, I am wondering whether the authors applied their method to solve for the (hopefully small) distortions in their ophthalmoscope?*

We have not, although are planning to do this in future work as we compare performance on a variety of ophthalmic imaging systems.

*Line 439: It is not at all clear to me that the static and dynamic distortions can be treated independently; it seems likely to me that the presence of static distortions will introduce small errors in the estimation of the dynamic distortions, although these will probably be quite small for small movements, and slowly-varying static distortions. This could be explored in simulation.*

Regrettably we are not able to offer a more formal explanation, lacking a mathematical proof. A proof would be non-trivial considering the arbitrary nature of distortions that can be handled by both algorithms, especially with dynamic distortion changing randomly from frame to frame. Despite the lack of an analytical proof, the empirical success of the results is very encouraging.

In the discussion we had already added the following rationale as to why the proposed method of correcting dynamic and then static distortion should be successful. This is the extent of the argument that we can provide; we have further added the comment by the reviewer regarding further simulation work to be done, which would be outside the scope of the present manuscript.

(Line 668)

“It may at first appear surprising that the output of the previously described dynamic correction procedure would combine well with the newly proposed static correction procedure. However, this marriage is assured by the large number of frames that we included within each cluster. This means that the interaction occurring between systematic and dynamic warp within one cluster should be expected to be nearly identical to any such interaction taking place in another cluster. In other words, the residual static error should not differ appreciably between clusters, given the large number of frames within each cluster. This satisfies the conditions for successful application of the DIOS procedure (a distortion function that is common to all clusters), such that near-perfect correction should be expected. It was noteworthy, though, that the residual static correction obtained was not simply equal to the ground truth static correction applied. Further work should explore the interaction between systematic and dynamic sources of distortion, which is likely to depend on the degree and spatial frequency of each type of distortion.”

*Line 600: “The method is blind to the absolute value of the distorting function...” – I think that “absolute value” is not the best term to use here. A constant translational shift is given here as an example, but isn’t that in fact the only component of distortion that falls into this category? I would rephrase it to say that the method is blind to prismatic shifts, or translations that are constant over the whole image. On second thought, I think that this also includes uniform magnification/minification; if this is true, then why not mention it as well?*

The reviewer’s intuition is correct on both fronts (prismatic shifts being the only example, and the method being blind to image magnification). We have re-worded/added as follows: (Line 625)

“… The method is blind to the absolute value of the distorting function, that is, to a fixed prismatic shift applied to all images. Such shifts merely move the image rather than producing distortion. The method would also be blind to uniform scaling (magnification) of the entire image set, unless the true amount of eye movement is known.”

*Lines 605-611: I'm not sure what is meant by "'islands' of connected pixels". I think what might be meant here is that however many images are collected, there need to be some that place a register-able feature at each image location. Obviously if the target (retina) has featureless regions, then there will be nothing to register and therefore no data to input to the algorithm. But with normal eyes, I don't see how this would ever arise in practice?*

We have added further clarification as follows: (Line 633)

“Where this is not the case, there are effectively “islands” of connected pixels produced within the image. A simple example where this occurs would be when the translation between images is vertical only, as in Fig 4; the “match” for each pixel will then always be found above or below it, but never to the right or left. This means that each column of the image effectively becomes an independent system of equations to solve. Because an arbitrary offset must be set for each system of equations (as the algorithm only solves for relative differences), these islands will invariably end up at different absolute displacement values, producing a “bumpy” appearance to the solution.”

*line 618: Another factor that could produce intensity differences between frames is vignetting produced in certain poses of the eye.*

This is true and we have now added this as follows: (Line 649)

“Such variations could be caused by factors such as light-evoked fluctuations in reflectance, the oxygenation state of blood, shot noise, non-uniformity of illumination, vignetting, etc.”

*lines 622-634: The paragraph discusses a two-step procedure for dealing with simultaneous static and dynamic distortions. It is not clear to me that static distortions won't introduce biases into the solution for the dynamic distortions, and that these biases won't in turn produce error in the DIOS process.*

In the next paragraph, our best justification for the success of the dynamic-and-then-static approach is given. We have quoted it already above in response to the reviewer’s earlier comment on the same issue. We quote it here again for convenience: (Line 668)

“It may at first appear surprising that the output of the previously described dynamic correction procedure would combine well with the newly proposed static correction procedure. However, this marriage is assured by the large number of frames that we included within each cluster. This means that the interaction occurring between systematic and dynamic warp within one cluster should be expected to be nearly identical to any such interaction taking place in another cluster. In other words, the residual static error should not differ appreciably between clusters, given the large number of frames within each cluster. This satisfies the conditions for successful application of the DIOS procedure (a distortion function that is common to all clusters), such that near-perfect correction should be expected. It was noteworthy, though, that the residual static correction obtained was not simply equal to the ground truth static correction applied. Further work should explore the interaction between systematic and dynamic sources of distortion, which is likely to depend on the degree and spatial frequency of each type of distortion.”

*The way that I conceptualize the problem of dynamic distortion is that there is a true underlying undistorted image, with N_rows x N_cols scalar parameters (larger than a single acquired image, as this represents the correctly registered mosaic of all of the images), and three pose parameters for each frame, parameterizing the eye pose (rotation). Each image contributes M_rows x M_cols data points (the pixel intensities in a single acquired frame), and 3 additional unknowns (the pose parameters). So it is clear that as we add images, the number of inputs grows much faster than the number of unknowns. But I don't know of a practical way to perform the optimization to obtain the solution (although there certainly may be one). Existing methods might provide a good initial solution that could be further refined. Incorporating static distortion into this model adds an additional 2 x M_rows x M_cols parameters (the distortion translation vector for each pixel), but as this is the same for every frame it does not increase as frames are added, it just makes the optimization a little more complicated. Even if it is too slow to be useful in practice, if such a method could be used to generate a solution, I would have more confidence in it than sequential optimization of sub-problems, and it would be.*

We note the reviewer’s proposed framework for a more satisfying approach to the problem of combined dynamic and static distortion. We are not sure that it would work as stated, as the number of unknowns does grow with each frame because in the dynamic case, distortion may be considered essentially random on each frame. The method that we developed previously (Bedggood and Metha 2017) and used again here was to exploit the zero-mean nature of stochastic variations in order to infer the dynamic distortions. That allowed us to circumvent this problem of an ever-increasing number of unknowns. I do not think that we can include the ideas of the reviewer in the paper without any more concrete idea of how it should all work. However, we hope that the reviewer would not mind, given the insight he has shown into the problem and the fact that he has not completed the review anonymously, if we are to contact him in the future to explore these ideas further.

*The problem discussed in the preceding paragraph is mentioned by the authors themselves at line 643, where they say, "It is noteworthy that although [sic] the residual static correction obtained was not simply equal to the underlying static correction applied." I'm not sure what this means, is the "underlying static correction applied" the same as the synthetic distortion applied, i.e. ground truth?*

We have corrected the typographic error noted and replaced the term “underlying” with “ground truth” (Line 676):

“… It was noteworthy, though, that the residual static correction obtained was not simply equal to the ground truth static correction applied.”

*Lines 688-693: The authors discuss various ways to make the computation of the solution more efficient. One approach that they may wish to consider is a progressive "coarse-to-fine" solution, where the distortion map is built up from progressively higher frequency components, kind of like solving for the Fourier components of the distortion map sequentially. The advantage is that the lower frequency components might be solved for using subsampled data (i.e., a "pyramid" approach), reducing the amount of computation required. This suggestion is close to the approach suggested at line 690, where they suggest down-sampling by "binning."*

We thank the reviewer for this suggestion, and have noted it in the following change: (Line 729)

“Another option still could be a pyramid approach, where lower frequency information is solved first, from sub-sampled data, before turning to progressively higher frequency information sampled at greater resolution.”
